# Notes and News

**Published:** 1894-07-07

**Authors:** 


### Notes and News.

Collected and Communicated.

MEETINGS OF THE WEEK.

Seamen's Hospital, Greenwich, Quarterly General Court, 24, Budge Row, Friday, July 13th, at 3 p.m.

Mr. Edwin T. Hall, whose plan was placed first by Mr. Waterhouse in the recent competition, has been appointed architect to the Park Hospital by the Metropolitan Asylums Board, and will proceed to erect the hospital from his plans forthwith.

Dr. Joseph Coats has been elected to the Chair of Pathology at the University of Glasgow, Professor Thaddaus, of Krakau, has been elected Academic Senate Rector of that University for 1894-95, and Dr. Joseph Fódor has been made Rector Magnificus of Buda-Pesth, where he is Professor of Hygiene.

At the recent ceremony of Inauguration of the Faculties at Caen, foreign universities were well represented. Oxford and Cambridge each sent three representatives to this interesting ceremony, which restored the ancient University of Caen, founded in 1432 by Charter granted by the Duke of Bedford on behalf of Henry VI. of England.

Various arrangements are being made to facilitate a visit to the Congress of Hygiene and Demography. Amongst the programmes offered to visitors is a tour, to cost only £20, organised by the Rev. H. Lunn, M.D., respecting which information may be obtained from Mr. H. Bishop, 5, Endsleigh Gardens, N.W. The party will travel the outward journey together, but those forming it can return separately.

Mr. Haffkine's system of cholera inoculation has met with some remarkable instances of success in India. In a number of cases where some members have been inoculated and some not, those operated upon have escaped the disease, which attacked others of the same household. Mr. Haffkine has been invited to Madras to introduce a system so successfully tried in Calcutta.

A colony for epileptics is to be established in the State of New York in America, named after the late Oscar Craig, President for some years of the State Board of Charities. A tract of 1,875 acres of beautiful land in the Genesee Valley, near Mount Morris, in Livingston County, is to be secured. This tract is all in one piece, and is already provided with picturesquely-grouped buildings to the number of thirty-five, previously occupied by Shakers. The buildings are all to be on the village plan. A board of five managers have been appointed, one of whom is a specialist on nervous and mental diseases. A lady residing within a few miles of the colony as one of the managers, in order that the women and children and general housekeeping may be kept under constant surveillance. The managers serve without salary, and meet at the colony once or oftener monthly.

The managers of the Craig Colony for Epileptics may accept any bequests of persons interested in the welfare of epileptics, and it is believed that many charitable wealthy people will build cottages to bear their names and exist as lasting memorials to their desire to serve humanity in this wise. A medical superintendent, steward, matron, pathologist, nurses, school-teachers, teachers of various industries and arts, and so on, are to be appointed as needed; but the colony will not be ready probably to receive patients before the autumn of 1895. It is thought that the colony will ultimately number fifteen hundred to two thousand members. As soon as possible the six hundred epileptics in the county almshouses will be taken in charge. Later private patients will be received at prices corresponding to the accommodations asked for. It is sure to become self-supporting in the course of time, and to grow into an industrial and agricultural village that will more than rival the similar and famous colony at Bielefeld, Germany, upon which this is, to a certain extent, modelled.

The Earl of Meath presided at the third annual meeting of the British College of Physical Education, at Exeter Hall, on Saturday, June 30th. The secretary (W. J. Welch, junr.), having read the annual report, its adoption was moved by the chairman. Lord Meath said: Ladies and gentlemen, you have heard from the report that the British College has been actively engaged during the past year in carrying out, as far as opportunity and means allowed, its published programme. Of course it is not possible to achieve all we purpose until we have a habitat. During the past twelve months a great change has taken place in public opinion with regard to the whole question of physical education, and the people are now prepared to welcome any means of improving the health and physique of the race. It is therefore more than ever necessary that the teachers should be well trained. In most Continental cities this training is undertaken by the Government at its own expense; in England it is not so, and the B.C.P.E. is trying to supply the lack. Therefore the college confidently appeals to all interested in the welfare and prosperity of the British race for that financial support which will enable them to build an institution with class rooms, lecture hall, &c., so that they may be in a position to realise all their aims. Major-General Moberly, Dr. Savill (chairman of Council), and others also addressed the meeting.

